# Dr. Elizabeth Blackwell

**Published:** 1910-07

**Authors:** 


Dr. Elizabeth Blackwell, widely known in the practice of medicine in England, where she was born, and in the United States, where she practised several years, died at her home in Hastings, Eng., June 1, 1910, aged eighty-nine years. In early life she taught in Kentucky and South Carolina, later studying medicine in Geneva, N. Y., where she graduated in 1847. In 1851 she began practice in New York City, where she founded a hospital and medical school for women. She returned to England in 1859.

